# How Do Children Deal With Conflict? A Developmental Study of Sequential Conflict Modulation

**DOI:** 10.3389/fpsyg.2018.00766

**Published:** 2018-05-23

**Authors:** Silvan F. A. Smulders, Eric L. L. Soetens, Maurits W. van der Molen

**Affiliations:** ^1^Department of Psychology, University of Amsterdam, Amsterdam, Netherlands; ^2^Department of Psychology and Educational Sciences, Vrije Universiteit Brussels, Brussels, Belgium

**Keywords:** sequential conflict modulation, conflict adaptation, Simon task, S-R compatibility task, hybrid choice reaction/NoGo task, development

## Abstract

This study examined age-related differences in sequential conflict modulation (SCM), elicited in three tasks requiring the inhibition of pre-potent responses; a Simon task, an S-R compatibility (SRC) task and a hybrid Choice-reaction/NoGo task. The primary focus was on age-related changes in performance changes following a conflict trial. A secondary aim was to assess whether SCM follows different developmental trajectories depending on the type of conflict elicited by the tasks. The tasks were presented to three different groups of participants with an age range between 7- to 25-years—one group of participants for each task. For each task, the response-to-stimulus interval (RSI) was manipulated (50 vs. 500 ms) across trial blocks to assess time-dependent changes in conflict modulation. The results showed SCM for all three tasks, although the specific patterns differed between tasks and RSIs. Importantly, the magnitude of SCM decreased with advancing age, but this developmental trend did not survive when considering age-group differences in basic response speed. The current results contribute to the emerging evidence suggesting that patterns of SCM are task specific and were interpreted in terms of multiple bottom-up control mechanisms.

## Introduction

The focus of the current study is on sequential conflict modulation (SCM) that may arise on speeded response tasks. More specifically, the current interest is on performance changes on trials following a conflict trial. In the current study, conflict is elicited in different ways; that is, conflict between the desired response and a response elicited by a task-irrelevant stimulus feature, conflict between the desired response and an overlearned or natural response, or conflict between the execution or inhibition of the selected response. Our main aim is to assess developmental trends in SCM from childhood into young adulthood and to examine whether this trend depends on the specific type of conflict encountered by the participant and the time that elapsed after encountering the conflict.

The typical pattern observed in adult studies of SCM consists of a substantial reduction in the performance decrement on conflict trials when such a trial is preceded by another conflict trial relative to a non-conflict trial (Duthoo et al., 2014). The predominant interpretation of this SCM suggests that individuals utilize previous conflict information to optimize current conflict resolution (Botvinick et al., 2001). That is, individuals are inclined to expect that (non-) conflict will repeat on the upcoming trial (e.g., Gratton et al., 1992). When they expect a conflict trial to repeat they will up-regulate cognitive control facilitating the processing of relevant stimulus features and the activation of the appropriate response, thereby reducing the performance decrement associated with conflict trials. In contrast, when they expect a non-conflict trial to repeat they will down-regulate cognitive control allowing the processing of irrelevant stimulus features and reducing the threshold for activating the competing, incorrect response. Neurocognitive studies provided convincing support for this view (e.g., Kerns et al., 2004) and the collateral hypothesis of individual differences in top-down cognitive control (e.g., Egner, 2011; Wang et al., 2015).

Surprisingly, there is only a handful studies examining SCM in children. Collectively, these studies yielded the anticipated pattern of findings reported in the adult literature. That is, a sizeable reduction of the conflict effect on trials following a conflict trial relative to the conflict observed on trials following a non-conflict trial. Stins et al. (2007) presented 12-year-olds with two conflict tasks; a Simon spatial compatibility task and an Eriksen flanker task. Their results indicated that SCM was present on both the Simon and the Eriksen task. These findings suggest that the mechanisms involved in SCM are in place already in 12-year-olds. Iani et al. (2014) performed a similar study by presenting a Simon task to 1st and 2nd graders. They observed SCM in both groups whereas the size of conflict modulation did not discriminate between groups. This confirms that SCM is already present in young children. Ambrosi et al. (2016), who used three different tasks (i.e., an Eriksen task, a Simon task, and a version of a Stroop color-word task), to assess SCM in a group of 5- to 6-year-olds. The results reported by Ambrosi et al. (2016) showed a sizeable sequential conflict-modulation effect associated with the Simon and Stroop tasks but the effect was considerably less pronounced for the Eriksen task. The between-tasks differences suggest task-specific differences of post-conflict performance consistent with the idea that SCM is domain specific (Egner, 2008).

The studies reviewed above indicated that SCM is present already in young children, but they did not evaluate developmental trends in conflict modulation. Two studies examined age-related change in SCM on tasks eliciting a conflict between stopping and going. Huizinga and Van der Molen (2011) observed that choice reactions following a NoGo trial were considerably slower than choice reactions following another choice reaction. In addition, SCM decreased with advancing age from 7-year-olds to young adults but it is important to note that this developmental trend did not survive when controlling for group differences in basic response speed. Van de Laar et al. (2011) studied SCM using a stop-signal task. In this task, participants were required to respond to the direction of a left- or right-pointing arrow. On a small proportion of trials the color of the arrow changed just following its onset. The results indicated that responses following a successful inhibit on a stop-signal trial were slower than responses on choice trials following another choice trial. SCM showed a significant developmental decrease from the 8- to the 12-year-olds into young adults. Similar to the results reported by Huizinga and Van der Molen (2011), however, the developmental trend did not survive when correcting for group differences in basic response speed. Larson et al. (2012) used a standard Stroop task to examine SCM in two age groups; 8- to 11-year-olds and 19- to 30-year-olds. The results did yield SCM in both age groups but this effect did not discriminate between age groups. Araujo et al. (2015) examined SCM in participants with an age range between 4- and 24-years using a Go/NoGo and a Simon task. They observed SCM on both tasks and all age groups. In addition, it was found that the SCM effect on the Simon, but not the Go/NoGo task, decreased linearly with age. The decrease was interpreted to suggest that conflict monitoring increases with advancing age. Cragg (2016) presented an Eriksen task to three age groups (7-, 10-, and 20-year-olds). The data pattern that emerged from this study was similar to the one reported by Larson et al. (2012). SCM was present in all age groups but the size of this effect was similar across age groups. Waxer and Morton (2011) had three age groups (9- to 11-, 14- and 15-, and 18- to 25-years-olds) to perform on a version of a Dimensional Card Sorting Task, including interleaved congruent and incongruent trials. Their results yielded SCM in the adolescents and adults but not in the young children. Finally, Verbruggen and McLaren (2017) presented two age groups (children, aged 4–11 years, and young adults) a continuous action control task on which participants were required to move a cursor to a target location. On a minority of the trials (i.e., change trials) the location of the target changed while participants were moving the target. The results revealed that moving time was shortened on change trials preceded by another change trial but lengthened on no-change trials preceded by a change trial. The SCM effect did not discriminate between age groups. This finding was taken to suggest that the mechanisms involved in performance adjustments following conflict mature at a faster rate than top-down control mechanisms.

The pattern of results that seems to emerge from the above review examining SCM in children makes a couple of important points. First, most studies observed SCM to be present in young children. Secondly, only few studies examined developmental change in SCM but the outcomes of those studies are inconsistent. Thirdly, studies of SCM in children yielded substantial differences across tasks. The latter observation is consistent with results reported in the adult literature suggesting that SCM is domain specific rather than domain general (for a review Braem et al., 2014). Tasks differ in the type of conflict that they generate and different types of conflict may require separate modes of control (e.g., Fan et al., 2003; Funes et al., 2010). Accordingly, Egner (2014), in reviewing the available evidence, concluded that SCM involves a complex machinery of bottom-up and top-down modulatory influences, the exact implementation of which depends upon the specific conflict encountered. From a developmental perspective one might add that age-related changes in SCM are likely to depend upon the modes of cognitive control that are available to the child.

## The current study

The primary goal of the current study was to examine age-related change in SCM using three different conflict tasks sharing a common implementation format but varying in the type of conflict elicited by the task. That is, participants were asked to respond to colored left- or right-pointing arrows by depressing left- or right-hand response buttons depending upon the color and/or directional information provided by the arrows. The tasks were administered in separate experiments and in order to obtain a sufficient number of trials, experiments were done between rather than within groups. In the first experiment, different age groups performed on a version of a standard Simon spatial compatibility task. Participants responded to the color of the arrow while ignoring the direction indicated by the arrow. Because of the possible overlap between the response and the (irrelevant) directional information associated with the arrow stimulus, responses are relatively fast when the response and arrow direction are congruent and slow when they are incongruent (for a review, Lu and Proctor, 1995). In the second experiment, a stimulus-response compatibility (SRC) task was used in which there is overlap between the relevant stimulus and response set. Here, the color of the arrow defines the S-R mapping rule; one color signals that the direction of the arrow indicates the responding hand (compatible trials) while the other color signals that the opposite response should be executed (incompatible trials). Typically, using a blocked presentation of SRC, responses are much faster on compatible relative to incompatible trials (for a review Proctor and Reeve, 1990). However, the speed advantage on compatible trials disappears with a mixed presentation of SRC (e.g., Mansfield et al., 2012). In the third experiment, a hybrid Choice-reaction/NoGo task was used. In this task, left-pointing arrows in one color required a left-hand response while left-pointing arrows in the other color for response inhibition and, vice versa, right-pointing arrows in the one color required response inhibition while right-pointing arrows in the other color required a right-hand response. In this task, conflict is elicited by the automatic activation of the response indicated by the direction of the arrow and the need to suppress this response when the color of the arrow signals that a response to the arrow should be inhibited. This task involves a demanding conjunction analysis of relevant stimulus features (arrow direction and arrow color) and, thus, it can be anticipated that participants are prone to make a substantial amount of commission errors (e.g., McNab et al., 2008).

In view of the inconsistencies reported in the developmental literature, it would be difficult to formulate strong predictions. Several studies indicated that young children exhibit already SCM (Stins et al., 2007; Iani et al., 2014; Araujo et al., 2015; Ambrosi et al., 2016; but see Waxer and Morton, 2011). Accordingly, we anticipated SCM to occur in young children. On the hypothesis that SCM results from top-down measures relying on prefrontal control mechanisms (e.g., Botvinick et al., 2001; Kerns, 2006) and the literature indicating a protracted maturational course of these control mechanisms (e.g., Luna et al., 2010; Crone and Ridderinkhof, 2011; Fjell et al., 2012; Church et al., 2017), one would anticipate that SCM increases with advancing age (but see Araujo et al., 2015). This outcome is consistent with the results reported by Waxer and Morton (2011) who observed SCM in adolescents and adults but not young children. The pattern of a developmental increase in SCM is likely to depend on the time elapsing between successive trials. Thus, Notebaert et al. (2006) reported that the performance changes following conflict elicited by in a Stroop task did not occur in adults when the stimulus-to-response interval (RSI) was very short (i.e., 50 ms) while they were clearly present when RSI was lengthened to 200 ms (but see Egner et al., 2010, for contrasting findings using longer RSIs). This observation suggests that the control measures resulting in SCM require some time for appropriate implementation and it can be expected that young children need more time than adults (e.g., Smulders et al., 2016). To address this issue, we examined age-related change in SCM vis-à-vis the manipulation of RSI (either 50 or 500 ms between trial blocks).

At this point, it should be noted that an alternative view of sequential modulation assumes that it results from bottom-up influences rather than top-down control. More specifically, it has been argued that SCM results from the repetition of specific features across trial sequences (e.g., Mayr et al., 2003). Indeed, it has been observed that SCM occurs only, or at least more strongly, on trials repeating features of the immediately preceding trial (for a review Schmidt et al., 2015). Previously, we observed that young children are particularly sensitive to repetition priming, in particular when the time interval between successive trials is short (Smulders et al., 2005). Along these lines, it should be predicted that the size of the conflict modulation effect decreases with advancing age and, as a corollary, that the predicted developmental trend is steeper when RSI is short compared to long. It should be noted, however, that Waxer and Morton (2011) reported that stimulus repetition priming did not alter the age related trend in SCM. Likewise, Verbruggen and McLaren (2017) failed to observe a stimulus repetition effect in their developmental data.

When evaluating age-related change in SCM, it is important to consider that developmental outcome is task specific. Several studies reported findings suggesting the task specificity of SCM in children (Stins et al., 2007; Araujo et al., 2015; Ambrosi et al., 2016). These findings are consistent with the literature examining the domain-specificity of SCM (e.g., Braem et al., 2014, for a review). Hence, the type of conflict can be assumed to vary across the tasks used in the present study and, consequently, the manifestation of SCM might differ between tasks. Furthermore, given the task-specificity of SCM, it can be anticipated that the mechanisms mediating post-conflict performance mature at different rates.

A final issue to consider when evaluating age-related change in SCM is “proportionality.” The typical analysis of developmental trends in the speed of responding on different tasks or conditions is to submit the RTs derived from these tasks or conditions to repeated-measures ANOVA, which in its simplest form, involves two age groups (G1 and G2) and two manipulations (M1 and M2). A significant interaction between G and M is then of most interest, as this effect may suggest a specific age-group related effect related to one but not the other manipulation (e.g., young adults but not children delay their speed of responding on trials following a conflict trial but they do not on trials following a non-conflict trial). However, the Group × Manipulation interaction could be qualified by a significant main effect of Group, which is most likely the case in the present study given the age range under consideration. Given that there is a systematical relation between the group RTs and the difference in RT between manipulations, such that this difference increases with group RT then any manipulation that increases overall RT will result in a significant G × M interaction regardless of the processing involved (c.f. Salthouse and Hedden, 2002). Several methods can be used to address this issue (e.g., log-transformation of RT (e.g., Huizinga and Van der Molen, 2011; regression analysis (e.g., Hale et al., 1993). Here, we will assess developmental trends in SCM by submitting RTs first to regular repeated-measures ANOVA. The primary focus is then on interactions including Age group, Current trial, and Preceding Trial. Should such an interaction turn out to be significant we will then submit RTs to ANCOVA controlling for group differences in mean RT. When the interactions between Age group, Current trial and Preceding trial would remain significant we will conclude that the observed developmental trend in SCM is real rather than apparent. In that case, the developmental change in SCM is “disproportional;” that is, the developmental change in the SCM effect is process specific effect rather than being mediated by a global processing mechanism. On the other hand, when the interaction does not remain significant after controlling for age-group differences in mean RT, we will conclude that the mechanism involved in the SCM effect develops in concert with the other processing components included in the reaction process (Cerella and Hale, 1994; Kail and Salthouse, 1994). This outcome has been observed previously by Huizinga and Van der Molen (2011) and Van de Laar et al. (2011).

## Experiment 1: simon task and sequential conflict modulation

The current version of the Simon task required participants to respond to the color of left- or right-pointing arrows while ignoring the directional information associated with the arrows. On half of the trials the location of the response, right- or left-hand response, corresponds with the direction indicated by the arrow, right or left, whereas on the other half of the trials the location of the response does not correspond with arrow direction. The former type of trials is dubbed “congruent” and the latter “incongruent.” Numerous studies indicate that the task-irrelevant location information in a Simon paradigm has a relatively small but robust effect on the speed of responding—the speed of responding is delayed on incongruent relative to congruent trials (review in Lu and Proctor, 1995). This delay has been attributed to the need to suppress the pre-potent response toward the location of or indicated by the stimulus (e.g., Eimer, 1999; Miles and Proctor, 2012).

On the hypothesis assuming that the ability to inhibit a pre-potent response develops rapidly during childhood (e.g., Dempster, 1992; Van der Molen, 2000), one would be led to predict a decrease in the Simon effect with advancing age. However, the relatively scant developmental literature yielded inconsistent findings. Jerger et al. (1999) reported a developmental decrease of the Simon congruency effect using an auditory variety of the Simon task (e.g., responding to the speaker's gender while ignoring the speaker's location). Band et al. (2000) used an inter-modal Simon task requiring participants to respond to a visual stimulus while ignoring the location of a task-irrelevant auditory stimulus that was presented at different time intervals following the onset of the visual stimulus. The only developmental difference was a larger Simon congruency effect for auditory accessories presented at longer time intervals. Davidson et al. (2006) presented age groups with visual implementations of a Simon task differing in the type of visual stimulus (e.g., pictures, arrows, dots). They observed a developmental decrease in the Simon congruency effect for one task (presenting pictures) but not others (presenting arrows). Finally, Gathercole et al. (2014) performed a lifespan study (age range between 2- and 9-years) using a standard Simon task for adults and a child friendly version for children. This study showed that the Simon effect discriminated between age groups.

One aim of this experiment was to obtain a solid pattern of developmental change in the Simon congruency effect. The major goal of this experiment was, however, to replicate the recurrent finding of SCM in the Simon task (for a review, Kerns, 2006) and to assess whether SCM effect would change with advancing age. Recently, Ambrosi et al. (2016) observed a substantial Simon effect (48 ms) in 5-year olds and, most interestingly, the Simon effect was 105 ms on trials following a congruent trial whereas it was annihilated on trials following an incongruent trial. Thus, it was anticipated that a similar pattern would be observed here, at least for the long RSI. Moreover, the current results should reveal a developmental trend assuming that SCM is a manifestation of top-down cognitive control. Such a developmental trend should be absent for the short RSI as children, and possibly adults, would need more time for the instantiation of appropriate adjustment measures. We will further examine whether the developmental trend in SCM is disproportional or follows the group differences in basic response speed (Huizinga and Van der Molen, 2011). Finally, we will assess the contribution of bottom-up repetition priming influences in SCM and whether the impact of these influences changes across age groups (Waxer and Morton, 2011; Verbruggen and McLaren, 2017).

### Methods

#### Participants

Three age groups (*N* = 65) between 7- and 25-years of age participated in the experiment; a group of 21 young children between 7- and 9-years of age (*M* = 7.9 years; 12 girls), a group of 20 older children between 10- and 12-years of age (*M* = 11.4 years; 12 girls), and a group of 24 young adults between the ages of 18 and 25 (*M* = 21.0 years; 17 females) enrolled in the experiment. The children were selected with the help of their schools and with permission of their caregivers. All children had average or above average intelligence based on teacher reports. The young adults were undergraduate psychology students. They were recruited by flyers and received course credits for their participation. All participants reported to be in good health and had normal or corrected-to-normal vision. Informed consent was obtained from adult participants and primary caregivers of the children. The Ethical Review Board of the University of Amsterdam reviewed and approved all procedures.

#### Apparatus and stimuli

The experiment was run on 12-, and 15-inch screen computers and laptops. Stimuli were presented at the center of the screen, against a white background. The stimuli were left- vs. right-pointed arrows in red or blue and measuring 1.5 cm length and width. Participants viewed the monitor from a distance of 40–60 cm, and responded to the stimuli by pushing the “z” key with their left-index finger or the “/” key with their right-index finger. These keys are on the bottom row of a “querty” keyboard. The computer coded response accuracy and registered the speed of responding to the nearest millisecond. Reaction time (RT) was recorded as the time between stimulus onset and the moment that one of the response keys was switched. The response triggered the offset of the stimulus and started the response-to-stimulus interval (RSI), which was fixed at either 50 or 500 ms (between blocks manipulation).

#### Design and procedure

Participants performed a choice RT task in which they made a binary response to the color of the arrow while ignoring arrow directions. Red arrows required a left-hand response and blue arrows a right-hand response, or vice versa (counterbalanced across participants). An experimental session consisted of 10 experimental blocks; 5 short RSI blocks (50 ms) and 5 long RSI blocks (500 ms). Each RSI condition started with a 50-trials practice block, followed by the five experimental blocks consisting of 100 trials. The order of the RSI conditions was counterbalanced across participants.

### Results

For each age group and RSI, trials were sorted for Current trial congruence (congruent vs. incongruent current trials), and Preceding trial congruence (congruent vs. incongruent preceding trials).

#### Error rate

Errors and trials following an error were excluded from RT sorting. Error rates and median RTs are presented in Table 1, for each of the above trial categories. Error rates were square-root transformed prior to further analysis. The transformed error rates were subjected to ANOVA with Age group (3), as between-SS factor, and Current congruence (2), Preceding congruence, and RSI (2), as within-SS factors.

**Table 1:** Mean RT (ms; upper table) and Error Rate (%; lower table) (incl. SD) for each trial sequence, RSI, and age group (Experiment 1).

**Trial sequences**								
	**RSI-50 ms**				**RSI-500 ms**			
**Age group**	**C-C**	**C-IC**	**IC-C**	**IC-IC**	**C-C**	**C-IC**	**IC-C**	**IC-IC**
**RT/SD (ms)**								
7–9 years	558.30/78.94	723.08/82.98	642.79/77.73	606.93/79.76	528.04/45.43	671.18/54.85	599.73/55.41	679.44/53.53
10–12 years	529.30/44.32	651.73/77.30	588.01/68.18	616.61/60.34	487.50/63.04	625.95/45.10	525.33/49.51	623.56/59.61
18–25 years	435.38/57.21	509.34/59.31	446.47/62.68	479.57/64.69	362.52/45.01	448.50/55.65	392.88/45.49	417.64/53.97
**Errors/SD (%)**								
7–9 years	4.6/1.4	5.6/1.5	5.4/1.0	5.3/1.5	5.8/1.4	6.2/1.0	6.2/1.6	7.0/1.2
10–12 years	5.2/1.7	4.7/1.0	5.5/1.4	4.6/1.3	5.8/1.1	4.2/1.7	4.9/1.8	5.8/1.9
18–25 years	4.5/0.9	5.1/1.5	4.2/0.9	2.9/1.2	4.5/1.9	4.5/1.8	4.3/2.4	4.4/2.0

*C-C, current Congruent trial preceded by a Congruent trial; C-IC, current Incongruent trial preceded by a Congruent trial; IC-C, current Congruent trial preceded by an Incongruent trial; IC-IC, current Incongruent trial preceded by an Incongruent trial*.

Error rates were low (around 5.0%) and decreased with advancing age (5.8, 5.1, and 4.3% for young children, older children, and adults, respectively), *F*_(2, 62)_ = 6.19, *p* < 0.004, η^2^_*p*_ = 0.17. The Current congruency effect on error rate did not reach significance; 5.1 and 5.0% on congruent vs. incongruent trials, respectively, *p* > 0.35, and was not influenced by Preceding congruency trial, *p* > 0.92. All other effects did not reach significance, *p*s > 0.59. In order to rule out explanations in terms of speed accuracy trade-off, correlations between error rates and RTs were calculated by type of sequences. Correlations were negative (−0.40 ≤ *r*s ≤ −0.13 for 7–9 years; −0.22 ≤ *r*s ≤ −0.06 for 10–12 years; −0.21 ≤ *r*s ≤ −0.29 for adults), but did not appear to be significant, *p*s > 0.05.

#### Response speed

Median RTs were subjected to ANOVA with Age group (3), as between-SS factor, and Current congruence (2), Preceding congruence, and RSI (2), as within-SS factors. The speed of responding increased with advancing age, *F*_(2, 62)_ = 80.84, *p* < 0.001, η^2^_*p*_ = 0.72. Adults responded faster (*M* = 437 ms) than both older (*M* = 581 ms) and younger children (*M* = 626 ms). The RSI effect increased with advancing age; young children, 13 ms, older children, 31 ms, young adults, 62 ms, *F*_(2, 62)_ = 5.79, *p* < 0.005, η^2^_*p*_ = 0.16. The RTs revealed a pronounced Current congruency effect, *F*_(1, 62)_ = 1,527.80, *p* < 0.001, η^2^_*p*_ = 0.96. The speed of responding on incongruent trials was considerably slower than on congruent trials (*M* = 588 ms and *M* = 508 ms, respectively). Importantly, the Current congruency effect was altered significantly by Age group, *F*_(2, 62)_ = 41.89, *p* < 0.001, η^2^_*p*_ = 0.58. The Current congruency effect was smaller for adults (*M* = 55 ms) compared to the older (*M* = 97 ms) and young children (*M* = 88 ms), who did not differ significantly, *p* > 0.11. The Current congruency effect was larger for long compared to short RSIs; respectively, 95 vs. 64 ms. But this effect was observed only for children, *p*s < 0.001, not adults, *p* > 0.58.

**Figure 1:**
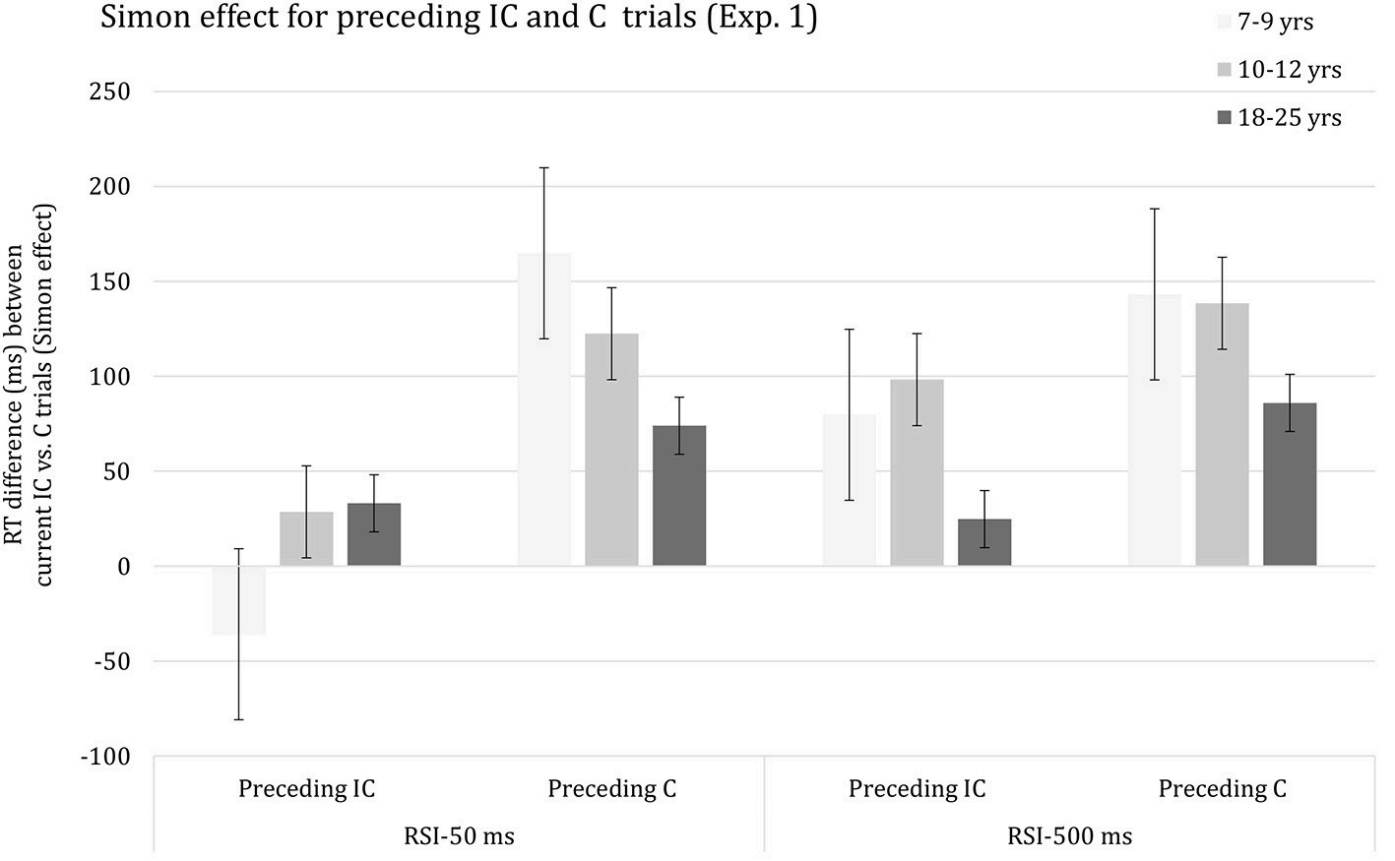
Reaction time difference (ms), including standard error bars, between current incongruent (IC) vs. congruent (C) trials (i.e., Simon effect) for preceding incongruent (IC) and congruent (C) trials, and for each age group and response-to-stimulus interval (RSI) condition.

As anticipated, the Current congruency effect was altered significantly by Preceding congruency, *F*_(1, 62)_ = 383.74, *p* < 0.001, η^2^_*p*_ = 0.86. The congruency effect was considerably larger on trials preceded by a congruent trial (*M* = 121 ms) relative to an incongruent trial (*M* = 38 ms). Importantly, the interaction between the effects of Current congruency and Preceding congruency was included in significant three-way interactions with the effect of Age group, *F*_(2, 62)_ = 34.21, *p* < 0.001, η^2^_*p*_ = 0.53 and RSI, *F*_(1, 62)_ = 36.79, *p* < 0.001, η^2^_*p*_ = 0.37, respectively. Finally, the effects of Age group, Current congruency, Preceding congruency and RSI were included in a complex higher-order interaction, *F*_(2, 62)_ = 24.52, *p* < 0.001, η^2^_*p*_ = 0.44, which is plotted in Figure 1. It can be seen that there is a sizeable Current congruency effect on trials following a congruent trial associated with both RSI 50 ms (Figure 1, left) and RSI 500 ms (Figure 1, right). The Current congruency effect is considerably smaller on trials following an incongruent trial for RSI 500 ms and is basically annihilated on trials following an incongruent trial for RSI 50 ms. The data suggest that the size of the SCM effect decreases with advancing age for both RSIs.

In order to decompose the complex higher-order interaction yielded by the omnibus ANOVA, follow-up analyses were then done for each RSI, separately. The ANOVA performed on the data associated with RSI 50 ms indicated that the interaction between the effects of Current congruency and Preceding congruency was significant in all three age groups (*p*s < 0.001). In addition, this interaction was included in a three-way interaction with Age group, *F*_(2, 62)_ = 43.47, *p* < 0.001, η^2^_*p*_ = 0.58, indicating that SCM decreased with advancing age, with each age group differing significantly from the other (*p*s < 0.002). In contrast, the analysis of the results associated with RSI 500 ms yielded a significant interaction between the effects of Current congruence and Preceding congruence in all three age groups (*p*s < 0.001), but the apparent age-related trend failed to reach significance (*p* > 0.16).

**Figure 2:**
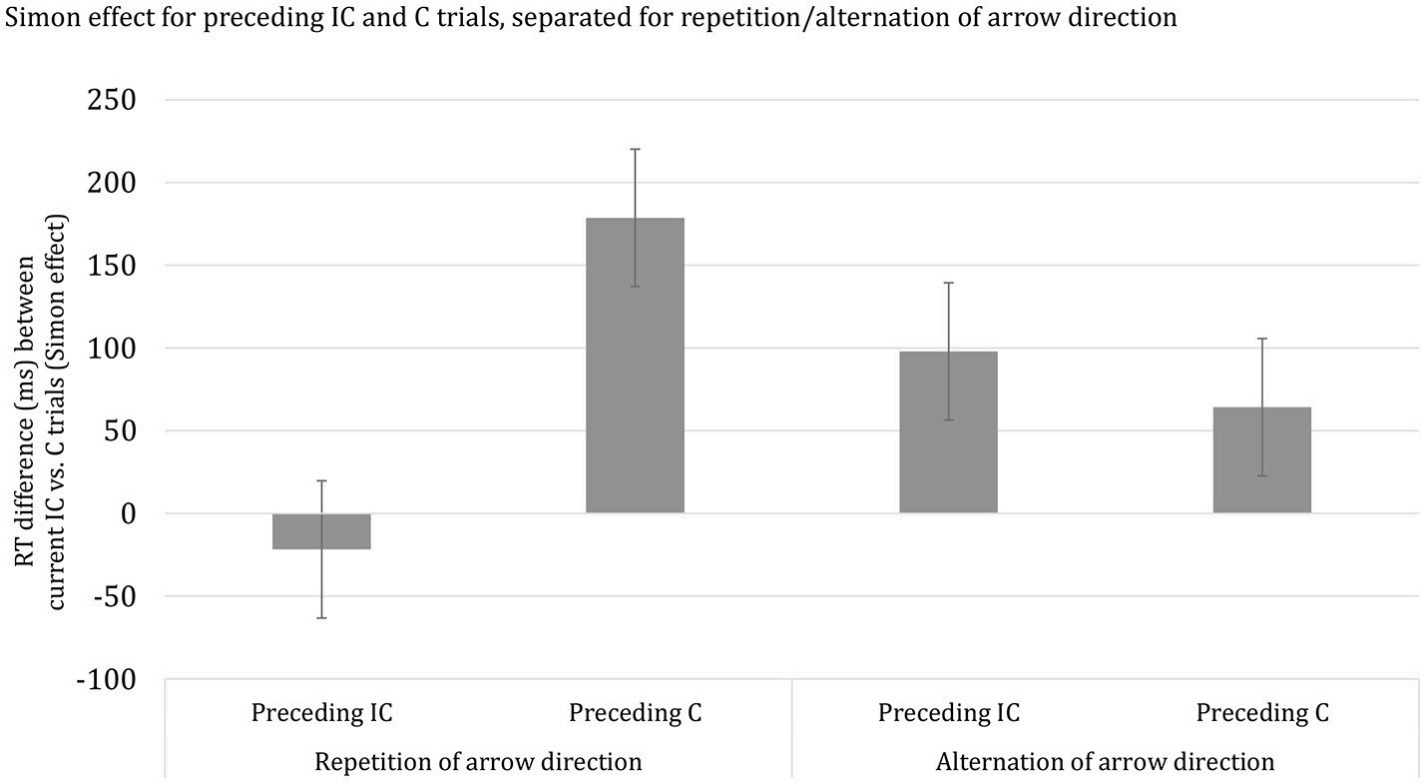
Reaction time difference (ms), including standard error bars, between current incongruent (IC) vs. congruent (C) trials (i.e., Simon effect) for preceding incongruent (IC) and congruent (C) trials, separated for repetition/alternation of arrow direction.

Finally, we considered the proportionality of the observed developmental trend in SCM and examined the potential influence of stimulus repetition priming. The analysis, using overall mean RT per participant as a covariate, revealed that the apparent age-related decrease in SCM was not disproportional. This finding indicates that the developmental trend in the SCM effect follows the overall trend in the speed of responding. Subsequently, we examined the influence of stimulus repetition priming. It should be noted, however, that the current dataset did not contain a sufficient number of trials for a full examination of the repetition-priming account of SCM, including stimulus and response sequences. Thus, we averaged data across the two RSIs and categorized trial sequences in terms of repetitions vs. alternations of arrow direction. Response repetitions or alternations were not considered. The ANOVA with Age group (3), as between SS-factor, Current congruency (2), Preceding congruency (2), and Priming (2), as within-SS factors yielded a highly significant interaction between the effects of Current congruency, Preceding congruency and Priming, *F*_(2, 62)_ = 418.63, *p* < 0.001, η^2^_*p*_ = 0.87. In Figure 2, it can be seen that SCM is prominently present for stimulus repetitions and virtually absent for alternations. Importantly, the higher-order interaction Current congruency, Preceding congruency, Priming, and Age group failed to reach significance, *p* = 0.14.

### Discussion

The current implementation of the Simon task required participants to respond to the color of left- or right-pointing arrows while ignoring the directional information of the arrow. In this version of the Simon task, the conflict is elicited by the location of the required response and the directional information associated by the arrow. Consistent with the literature (Vu and Proctor, 2004), the speed of responding was considerably slower on incongruent trials (with conflicting stimulus and response features) relative to congruent trials (without conflict). This pattern was observed for all three age groups, but the Simon effect was significantly larger in children compared to adults. This finding is consistent with previous developmental studies of the Simon effect (e.g., Jerger et al., 1999; Gathercole et al., 2014; Araujo et al., 2015; but see Band et al., 2000).

The size of the Simon effect was reduced considerably on trials following an incongruent relative to a congruent trial. This finding is in line with the adult literature on SCM using various versions of the Simon task (e.g., Stürmer et al., 2002; Kerns, 2006; Soetens et al., 2010; Duthoo et al., 2014, for a review). The results indicated that SCM was influenced by RSI. The results showed that the Simon effect following an incongruent trial was considerably reduced when RSI was 500 ms but it was basically annihilated when RSI was shortened to 50 ms. This finding stands in contrast with the results reported previously by Notebaert et al. (2006) who failed to observe SCM when RSI was 50 ms. The apparent discrepancy between the current findings and the results reported previously by Notebaert et al. (2006) could be due to the use of different tasks. Notebaert et al. (2006) examined the temporal dynamics of SCM using a version of a Stroop task, whereas a Simon-task was used in the present Experiment. In this regard, the current findings would add to the literature emphasizing the domain specificity of conflict-adaptation effects (e.g., Braem et al., 2014).

The reduction of the Simon effect following an incongruent trial was observed for all three age groups. In addition, the size of SCM decreased with advancing age for the short but not long RSI. Moreover, the age-related trend did not survive when controlling for group differences in basic response speed. The age-proportional trend associated with an RSI of 50 ms presents a challenge to developmental notions of SCM governed by top-down control measures (e.g., Larson et al., 2012; Cragg, 2016) implemented by late maturing prefrontal brain regions (Anderson, 1998; Luciana and Nelson, 2002; Romine and Reynolds, 2005; Huizinga et al., 2006; Best and Miller, 2010; Tamnes et al., 2010; Albert and Steinberg, 2011; Best et al., 2011; Hughes, 2011; Lyons and Zelazo, 2011; Vuontela et al., 2013). In contrast, the current results suggest that SCM results from bottom-up repetition priming rather than top-down cognitive control. The analysis taking stimulus repetition (i.e., arrow direction) into account revealed that SCM was clearly present for stimulus repetitions but virtually absent for stimulus alternations. This observation is consistent with previous findings using a Simon task (e.g., Nieuwenhuis et al., 2006). We had anticipated that stimulus repetition priming would be more pronounced for young children compared to adults (e.g., Smulders et al., 2005) but current data failed to reveal significance. It should be noted, however, that our prediction was based on previous findings focusing on response priming. Leaving this qualification aside, the current results are most readily explained in terms of bottom-up influences on SCM.

## Experiment 2: S-R compatibility (SRC) task and SCM

In the present version of the SRC task, the stimuli were identical to those used in the previous experiment. Participants were asked to respond to the direction of the central arrow stimuli. The arrows were presented in two different colors; one color instructed participants to make a spatially compatible response (i.e., a left-hand response to a left-pointing arrow and a right-hand response to a right-pointing arrow) whereas the other color of the arrows instructed participants to make a spatially incompatible response to the direction of the arrow (i.e., a right-hand response to a left-pointing arrow and a left-hand response to a right-pointing arrow. Importantly, arrow color was mixed within trial blocks. The mixing of compatible and incompatible trials has been observed to annihilate the response speed advantage of compatible over incompatible trials when presented in pure blocks (e.g., SHAFFER, 1965; Van Duren and Sanders, 1988; Heister and Schroeder-Heister, 1994; De Jong, 1995; Stoffels, 1996; Christensen et al., 2001; Proctor and Vu, 2002; Vu and Proctor, 2004). More specifically, compatibility mixing reduces the speed of responding on compatible trials relative to blocked presentation, whereas presentation mode has only a minor effect on the speed of responding on incompatible trials. This pattern has been taken to suggest that compatibility mixing induces a strategic bias toward incompatibility resulting in an active suppression of the compatible mapping rule, thereby reducing the SRC effect on the speed of responding (e.g., De Jong et al., 1994).

Developmental studies examining spatial SRC effects are few and far between. Early studies by Clark (1982) and Ládavas (1990) showed a developmental decrease in the SRC effect on the speed of responding. Van der Wildenberg and Van der Molen (2004) reported a similar pattern that was interpreted to suggest that children experience greater difficulty than adults in inhibiting the over-learned directional response to the stimulus. Other studies, however, reported developmental stability rather than age-related change in the SRC effect. Wright and Diamond (2014), for example, examined SRC effects across a limited age range (from 6- to 10-years) and observed that for all ages the speed of responding was considerably faster on compatible relative to incompatible trials. Casey et al. (2002) reported that the cost of an incompatible relative to a compatible mapping did not differ between a child group (7- to 11-years) and a group of young adults. Similarly, Dornier and Meany (2003) reported a pronounced SRC effect that did not change with advancing age. At this point, it is difficult to provide a ready interpretation of the apparent inconsistencies between studies. To date, there is only one developmental study in which SRC was manipulated between and within trial blocks (Crone et al., 2004). This study examined age-related change in the flexible use of SRC mappings in three different age groups; 8-year-olds, 11-year-olds, and young adults. The results showed that the interaction between trial block (pure vs. mixed) and SRC mapping (compatible vs. incompatible) did not vary across age groups.

The goal of this experiment was to examine developmental change in SCM using an SRC task with a mixed presentation of compatible and incompatible trials. Consistent with previous studies, we anticipated that the typical SRC pattern associated with pure blocks (i.e., slower responses on incompatible relative to compatible trials) would be greatly reduced, or even absent, when using mixed SRC blocks (e.g., Van Duren and Sanders, 1992; Stoffels, 1996). When examining trial sequence effects, we predicted obtaining a greatly reduced or even reversed SRC effect on trials following an incompatible trial relative to a compatible trial (e.g., Jennings et al., 2002; Mansfield et al., 2012). On the hypothesis that the reduction of the SRC effect following an incompatible trial reflects top-down cognitive control (De Jong, 1995; Jennings et al., 2002; Mansfield et al., 2012), we predicted that the reduction of the SRC effect following an incompatible trial would increase with advancing age given the protracted developmental course of brain regions implicated in cognitive control (Luna, 2009; Diamond, 2011; Munakata et al., 2012; Hsu and Jaeggi, 2014; Zanolie and Crone, 2018). Similar to the previous experiment, we will assess the proportionality of the age-related change in SCM (e.g., Huizinga and Van der Molen, 2011) and, in addition, examine the potential influence of stimulus repetition priming (e.g., Waxer and Morton, 2011).

### Methods

#### Participants

Participants (*N* = 64) were recruited from three age groups. There were two groups of children; 23 children between 7- and 9-years of age (*M* = 8.2 years; 14 girls) and 21 children between 10- and 12-years of age (*M* = 11.7 years; 11 girls). Finally, a group of 20 young adults between the ages of 18 and 25 (*M* = 22.3 years; 15 females) enrolled in the experiment. The children were selected with the help of their schools and with permission of their caregivers. All children had average or above average intelligence based on teacher reports. The young adults were undergraduate psychology students. They were recruited by flyers and received course credits for their participation. All participants reported to be in good health and had normal or corrected-to-normal vision. The Ethical Review Board of the University of Amsterdam reviewed and approved all procedures.

#### Apparatus and stimuli

All details concerning the apparatus and stimuli were the same as in Experiment 1.

#### Design and procedure

Participants were asked to respond to the direction indicated by blue arrows and in the opposite direction to red arrows, or vice versa (counterbalanced across participants). All other design details were the same as in Experiment 1.

### Results

For each age group, trials were sorted for Current compatibility (compatible vs. incompatible current trials), Preceding compatibility (compatible vs. incompatible preceding trials), and RSI (50 vs. 500 ms). Errors and trials following an error were excluded from RT sorting. Error rates and median RTs are presented in Table 2, for each of the above categories. Error rates were square-root transformed prior to analyses.

**Table 2:** Mean RT (ms; upper table) and Error Rate (%; lower table) (incl. SD) for each trial sequence, RSI, and age group (Experiment 2).

**Trial sequences**								
	**RSI-50 ms**				**RSI-500 ms**			
**Age group**	**C-C**	**C-IC**	**IC-C**	**IC-IC**	**C-C**	**C-IC**	**IC-C**	**IC-IC**
**RT/SD (ms)**								
7–9 years	836.71/108.29	1,142.96/94.47	1,143.62/67.33	887.81/97.80	773.83/95.64	1,007.30/86.09	1,062.33/82.36	797.29/94.23
10–12 years	745.28/105.71	956.03/94.86	988.34/98.98	768.70/89.11	614.56/59.35	821.95/97.95	854.45/123.03	692.09/91.20
18–25 years	651.29/66.01	878.46/84.16	875.96/102.50	674.67/68.01	511.20/44.23	676.27/62.46	688.81/63.52	541.15/57.89
**Errors/SD (%)**								
7–9 years	6.6/1.8	8.1/1.4	6.2/1.7	7.1/1.6	8.0/2.0	7.9/1.8	8.2/1.4	7.8/1.7
10–12 years	7.1/0.6	7.0/0.5	7.9/0.8	7.0/0.6	7.9/0.6	6.3/0.7	6.5/0.7	8.1/0.5
18–25 years	5.9/1.1	6.3/1.1	5.8/1.1	5.3/1.0	5.8/0.9	6.0/0.9	5.2/1.0	6.1/1.1

*C-C, current Compatible trial preceded by a Compatible trial; C-IC, current Incompatible trial preceded by a Compatible trial; IC-C, current Compatible trial preceded by an Incompatible trial; IC-IC, current Incompatible trial preceded by an Incompatible trial*.

#### Error rate

The transformed error data were subjected to ANOVA with Age Group (3), as between-SS factor, and Current compatibility (2), Preceding compatibility (2), and RSI (2) as within-SS factors. In Table 2 it can be seen that error rates are relatively low (≤8.2%). Error rates decreased with advancing age (from 7.5% in young children, to 7.2% in older children and 5.8% in adults), *F*_(2, 61)_ = 12.53, *p* < 0.001, η^2^_*p*_ = 0.29. Error rate was only slightly higher on incompatible (*M* = 6.9%) than compatible trials (*M* = 6.7%), *F*_(1, 61)_ = 15.22, *p* < 0.001, η^2^_*p*_ = 0.20, and this effect differed across age groups, *F*_(2, 61)_ = 22.46, *p* < 0.001, η^2^_*p*_ = 0.42. Adults and young children made more errors on incompatible than compatible trials (5.7 vs. 5.9% for adults, 7.2 vs. 7.8% for young children, *p*s < 0.001). Older children showed the opposite pattern (7.3 vs. 7.0%), *p* < 0.0.16. The interaction between RSI and Current compatibility was not significant, *p* > 0.20, but both effects were included in a complex higher-order interaction; Age group × Current compatibility × Preceding compatibility × RSI, *F*_(2, 61)_ = 32.99, *p* < 0.001, η^2^_*p*_ = 0.52. Separate ANOVAs were then done on the data associated with RSI 50 ms and RSI 500 ms to decompose the complex interaction. The ANOVA done on the RSI 50 ms data showed that the interaction between Age group, Current compatibility and Preceding compatibility was not significant, *p* = 0.31. The ANOVA done on the RSI 500 ms data yielded a significant three-way interaction, *F*_(2, 61)_ = 99.25, *p* < 0.001, η^2^_*p*_ = 0.77. The interaction between Current and Preceding compatibility was significant for adults, *p* < 0.001, and older children, *p* < 0.001, but not for the youngest children, *p* > 0.16.

In the bottom panel of Table 2, it can be seen that error rate in young children and adults, but not older children, is somewhat lower on incompatible trials preceded by another incompatible trial relative to incompatible trials followed by a compatible trial when RSI is short. This pattern changes into its opposite when RSI is long (i.e., both young children and adults did not show any error rate differences between both trial sequences, but older children made more errors on IC-IC as compared to C-IC sequences). Finally, in order to rule out explanations in terms of speed accuracy trade-off, correlations between error rates and RTs were calculated in the same way as in Experiment 1. The correlations were weak and mostly negative (−0.03 ≤ *r*s ≤ +0.31 for 7–9 years; −0.16 ≤ *r*s ≤ +0.04 for 10–12 years; −0.18 ≤ *r*s ≤ +0.24 for adults), but did not appear to be significant, *p*s > 0.05.

#### Response speed

Median RTs were subjected to ANOVA with Age group (3), as between-SS factor, and Current compatibility (2), Preceding compatibility (2), and RSI (2), as within-SS factors. The speed of responding increased with advancing age, *F*_(2, 61)_ = 190.72, *p* < 0.001, η^2^_*p*_ = 0.86. Adults responded faster (*M* = 687 ms) than older (*M* = 805 ms) and younger children (*M* = 956 ms). Responses were faster to a long RSI (*M* = 753 ms) compared to a short RSI (*M* = 879 ms), *F*_(1, 61)_ = 163.04, *p* < 0.001, η^2^_*p*_ = 0.73. The RSI effect was stronger in children (*M* = 166 and *M* = 119 ms for young and older children, respectively) compared to young adults (*M* = 93 ms), *F*_(2, 61)_ = 4.70, *p* < 0.013, η^2^_*p*_ = 0.13.

As anticipated, there was little difference in the speed of responding between compatible (*M* = 801 ms), vs. incompatible trials (*M* = 831 ms), *p* > 0.16. The apparent elimination of the typical SRC effect (i.e., slower responses on incompatible than compatible trials), due to the mixed presentation of SRC mappings, was present for each age group; i.e., the main effect of SRC did not interact with the effect of Age group (*p* > 0.90). Importantly, there was a highly significant interaction between the effects of Current compatibility and Preceding compatibility, *F*_(1, 61)_ = 565.64, *p* < 0.001, η^2^_*p*_ = 0.90. Compatible responses were considerably faster than incompatible responses when the preceding trial was compatible (*M* = 689 ms vs. *M* = 914 ms, respectively). When the current trial followed an incompatible trial, however, the SRC effect changed into its opposite (*M* = 936 ms for compatible trials vs. *M* = 727 ms for incompatible trials).

The interaction between Current and Preceding compatibility was altered by RSI, *F*_(1, 61)_ = 9.70, *p* < 0.003, η^2^_*p*_ = 0.14. For RSI 50 ms, the SRC effect was 248 ms for trials preceded by a compatible trial and −226 ms when the preceding trial was incompatible. For RSI 500 ms, these values were, respectively, 202 and −192 ms.

**Figure 3:**
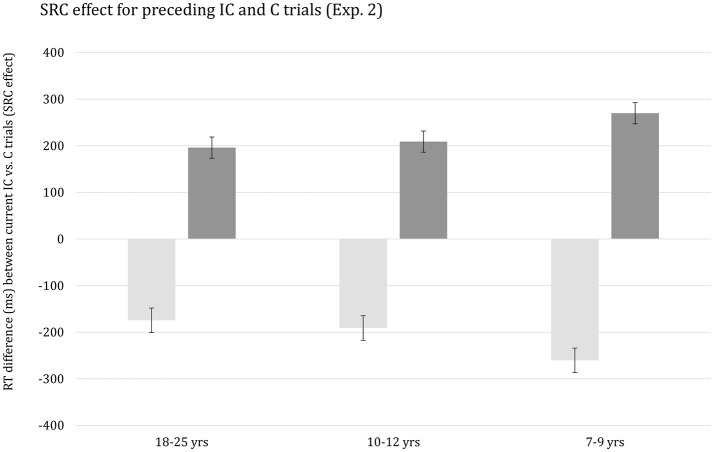
Reaction time difference (ms), including standard error bars, between current incompatible vs. compatible trials (i.e., SRC effect) for preceding incompatible (IC) and compatible (C) trials, and for each age group.

There was a highly significant interaction between the effects of Current compatibility, Preceding compatibility, and Age group, *F*_(2, 61)_ = 7.47, *p* < 0.001, η^2^_*p*_ = 0.20. This interaction is plotted in Figure 3. The figure indicates that SRC on the preceding trial alters the SRC effect on the current trial and this effect is stronger for the youngest children relative to the two older age groups. It should be noted, however, that the higher-order interaction did not survive when overall mean RT per participant was included as covariate, *p* > 0.54. Finally, the higher-order interaction including RSI was not significant (*p* > 0.63).

As in the previous experiment, we evaluated the potential influence of bottom-up stimulus priming on the sequential modulation of compatibility effects. We averaged data across the two RSIs and categorized trial sequences in terms of repetitions vs. alternations of arrow direction. The factor Priming, as within-SS factor, was then included in an ANOVA with Age group (3), as between-SS factor), Current compatibility (2), Preceding compatibility and Priming (2), as within-SS factor. The ANOVA revealed that the interaction between the effects of Current compatibility, Preceding compatibility and Priming was not significant, *p* = 0.19. The higher-order interaction including Age group also failed to reach significance, *p* = 0.10.

### Discussion

The implementation of the current version of the SRC task was similar to the Simon task, in that participants were asked to respond to colored arrows, but the important difference is that now both the color and direction of the arrow determine the response. Conflict is then elicited when color and direction are associated with opposite responses. On compatible trials, the color of the arrow indicates that a response is required in the direction of the arrow whereas on incompatible trials the color indicates that the opposite response should be executed. The adult literature indicates that on pure blocks the speed of responding is considerably slower on incompatible than compatible trials (Kornblum et al., 1990), whereas mixing trials may result in the annihilation of the SRC effect (e.g., Van Duren and Sanders, 1992). The current results are consistent with the literature in showing that mixing compatibility resulted in the overall elimination of the SRC effect (e.g., Van Duren and Sanders, 1992; Stoffels, 1996). More specifically, however, the results showed that, consistent with previous studies (e.g., Jennings et al., 2002; Mansfield et al., 2012; but see De Jong, 1995), the typical SRC effect (i.e., slower responses on incompatible relative to compatible trials) seen on trials following a compatible trial turned into its opposite on trials following an incompatible trial. It has been suggested that the reversal of the SRC effect on trials following an incompatible trial results from a preparatory bias for the incompatible mapping (e.g., Jennings et al., 2002). The preparatory bias consists of the suppression of the compatible mapping that has to be released when a compatible, not an incompatible, mapping is called for (De Jong, 1995). This preparatory bias has been interpreted in terms of proactive control; that is, a willful strategy facilitating incompatible mappings (Mansfield et al., 2012).

On the hypothesis that young children are less able or inclined to adopt a top-down strategy in handling cognitive conflict (e.g., Munakata et al., 2012; Chevalier et al., 2014), we anticipated that SCM of the speed of responding on the SRC task would be less manifest in children than adults. The results were opposite. If anything, SCM was stronger, not weaker, in children although it should be noted that the differences between age groups lost significance when controlling for group differences in basic response speed. The current failure to obtain a disproportional developmental trend in SCM on the SRC task may present a challenge to notions that proactive control is a key factor in producing this pattern.

A second challenge to the idea that SCM results from top-down cognitive control is presented by the current observation that this pattern is less rather than more manifest for the longest RSI. SCM of the SRC effect was more pronounced for the short relative to the long RSI. The current data pattern is in conflict with notions suggesting that the implementation of control operations following conflict is effortful and time consuming (e.g., Notebaert et al., 2006). On the other hand, however, we observed that stimulus priming failed to significantly alter the sequential modulation pattern on the SRC task, in contrast to the findings that emerged from a similar analysis of the speed of responding on the Simon task. In conclusion, the current findings seem to indicate that SCM on the SRC task are neither consistent with an interpretation in terms of top-down cognitive control nor with an interpretation attributing sequential modulation to bottom-up stimulus priming. But again, it should be noted that we considered only stimulus, not response, repetition trial sequences.

## Experiment 3: hybrid choice reaction/NoGo task and SCM

We used a hybrid Choice-reaction/NoGo task task derived from Van Boxtel et al. (2001). In this task, a left- or right-pointing arrow is presented in red or blue color. The combination of arrow direction and color determines whether a response should be executed or withheld. Thus, a red and left-pointing arrow may require a left-hand response while a red and right-pointing arrow requires response inhibition or a blue and right-pointing arrow may require a right-hand response while a blue and left-pointing arrow may ask for response inhibition. Adult findings derived from a variety of Go/NoGo tasks showed that the speed of responding is delayed on Go trials following a NoGo trial relative to a Go trial (e.g., Rieger and Gauggel, 1999; Hoffmann et al., 2003; Rieger et al., 2003; Schuch and Koch, 2003; Kleinsorge and Gajewski, 2004).

In the developmental literature, Go/NoGo tasks have been used widely to examine age-related changes in the ability to inhibit pre-potent responses (e.g., Luria, 1961; Levin et al., 1991; Casey et al., 1997; Durston et al., 2002; Jonkman et al., 2003; Brocki and Bohlin, 2004; Span et al., 2004; Johnstone et al., 2007; Cragg and Nation, 2008; Garon et al., 2008; Hämmerer et al., 2010; Iida et al., 2010; Huizinga and Van der Molen, 2011). The results of most studies employing a Go/NoGo task converge on the conclusion that the ability to inhibit a pre-potent response develops rapidly during childhood and reaches mature levels when children enter the adolescent period (Van der Molen, 2000).

Huizinga and Van der Molen (2011) examined developmental change in the speed of responding on choice reaction trials when these trials were preceded by a NoGo trial vs. another choice reaction trial. They observed that choice reactions were significantly delayed when preceded by response inhibition on a NoGo trial relative to response execution on a choice-reaction trial. In one important respect, the current implementation of the hybrid Choice-reaction/NoGo task was different from the one used by Huizinga and Van der Molen (2011). That is, the current task required a demanding conjunction analysis of the direction and color information provided by the arrow stimulus in order to retrieve the appropriate response. The conjunction analysis may impose substantial demands on working memory. Given the protracted course of working-memory development (e.g., Huizinga et al., 2006), we assumed that the conjunction analysis would reduce the capacity young children have available for top-down cognitive control ensuring appropriate performance following conflict. On the hypothesis that working-memory demands and SCM may interact (e.g., Weldon et al., 2013; Gulbinaite et al., 2014), we anticipated to observe a pronounced upward trend in the SCM effect with advancing age. Similar to the previous experiments, we will examine whether the predicted developmental trend is disproportional and influenced by stimulus repetition priming.

### Methods

#### Participants

Participants (*N* = 66) were recruited from three age groups; between 7- and 25-years of age. There were two groups of children; 20 children between 7- and 9-years of age (*M* = 8.4 years; 16 girls) and 24 children between 10- and 12-years of age (*M* = 11.3 years; 13 girls). Finally, a group of 22 young adults between the ages of 18 and 25 (*M* = 21.8 years; 17 females) enrolled in the experiment. The children were selected with the help of their schools. All children had average or above average intelligence based on teachers reports. They received a small present for their participation. The young adults were undergraduate psychology students. They were recruited by flyers and received course credits for their participation. All participants reported to be in good health and had normal or corrected-to-normal vision. Informed consent was obtained from adult participants and primary caregivers of the children. The Ethical Review Board of the University reviewed and approved all procedures.

#### Apparatus and stimuli

All details concerning the apparatus and stimuli were the same as in Experiment 1.

#### Design and procedure

Participants performed a hybrid Choice-reaction/NoGo task. Red arrows pointing to the right required a right-hand response and blue arrows pointing to the left required a left-hand response. In order to elicit a conflict situation, participants should refrain from responding to blue arrows pointing to the right or red arrows pointing to the left. This set-up was counterbalanced across participants. On successful inhibits on NoGo trials, the stimulus was terminated after 3 s and stimulus offset initiated the RSI started with a delay of 3 s. The order of arrow directions and colors was pseudo-random. All other design and procedural details were the same as in the previous experiments.

### Results

For each participant, trials were sorted for Current Choice-reaction, Preceding Choice-reaction/NoGo (Choice reaction vs. NoGo responses) and RSI (50 vs. 500 ms). Errors and trials following an error were excluded from RT sorting. Median RTs and error rates (choice errors and commission errors) are presented in Table 3 for each of the above categories.

Table 3Mean RT (ms; upper table) and Error Rate (choice and commission %; lower table) (incl. SD) for each trial sequence, RSI and age group (Experiment 3).

**Table:** 

**Trial sequences**				
	**RSI-50 ms**	**RSI-3 s**	**RSI-500 ms**	**RSI-3 s**
**Age group**	**Choice Reaction–Choice Reaction**	**NoGo–Choice Reaction**	**Choice Reaction–Choice Reaction**	**NoGo–Choice Reaction**
**RT/SD (ms)**				
7–9 years	783.31/107.53	909.64/69.54	716.09/134.71	839.05/11.90
10–12 years	597.17/68.71	725.66/80.97	578.79/41.83	668.58/71.92
18–25 years	443.99/44.71	478.68/45.72	382.60/39.13	424.76/23/15

**Table:** 

**Trial sequences**								
	**Errors (choice)/SD (%)**				**Errors (commission)/SD (%)**			
	**RSI-50 ms**	**RSI-3 s**	**RSI-500 ms**	**RSI-3 s**	**RSI-50 ms**	**RSI-3 s**	**RSI-500 ms**	**RSI-3 s**
**Age group**	**Choice Reaction–Choice Reaction**	**NoGo–Choice Reaction**	**Choice Reaction–Choice Reaction**	**NoGo–Choice Reaction**	**Choice Reaction–Choice Reaction**	**NoGo–Choice Reaction**	**Choice Reaction–Choice Reaction**	**NoGo–Choice Reaction**
7–9 years	7.5/1.6	7.0/1.4	8.9/1.5	8.0/1.4	10.1/2.1	9.2/2.0	8.1/1.9	10.2/2.0
10–12 years	5.3/2.2	5.4/2.3	5.9/2.7	5.8/2.4	5.9/1.9	4.9/1.8	5.1/1.7	6.4/1.8
18–25 years	2.5/0.7	3.8/0.8	3.0/1.1	4.3/0.8	2.9/1.6	4.5/2.0	4.1/1.3	4.2/1.8

*Choice Reaction-Choice Reaction, current Choice Reaction trial preceded by a Choice Reaction trial; NoGo-Choice Reaction, current Choice Reaction trial preceded by a NoGo trial; Choice Reaction-NoGo, current NoGo trial preceded by a Choice Reaction trial; NoGo-NoGo, current NoGo trial preceded by a NoGo trial. RSI in Choice-reaction/NoGo task, 50 or 500 ms for Choice Reaction to Choice Reaction or Choice Reaction to NoGo sequences but for sequences starting with NoGo there was an inter-trial interval of 3 s*.

#### Error rate

Error rates were square-root transformed prior to analyses. Error rates were low (<6%) and there was not a correlation-pattern between error rate vs. RT indicating a speed-accuracy tradeoff (+0.04 ≤ *r*s ≤ +0.4 for 7–9 years; −0.03 ≤ *r*s ≤ −0.27 for 10–12 years; −0.08 ≤ *r*s ≤ −0.39 for adults; *p*s > 0.05). Error rates are presented for Current Choice-reaction/NoGo as a function of preceding Choice-reaction/NoGo in Table 3 for each age group and both RSIs.

#### Choice errors on choice-reaction trials

The transformed choice error rates were subjected to ANOVA with Age group (3), as a between-SS factor, and Preceding Choice-reaction/NoGo (2), and RSI (2), as within factors. Choice error rate decreased with advancing age; from 7.8, 5.6, and 3.4% for young children, older children, and adults, respectively, *F*_(2, 63)_ = 45.81, *p* < 0.001, η^2^_*p*_ = 0.59. There was a significant effect of Preceding Choice-reaction/NoGo, *F*_(1, 63)_ = 33.23, *p* < 0.001, η^2^_*p*_ = 0.35, but this effect was qualified by an interaction with Age group, *F*_(2, 63)_ = 95.04, *p* < 0.001, η^2^_*p*_ = 0.75. Adults made somewhat more choice errors following a NoGo trial (from 2.8 to 4.1%), *p* < 0.001, η^2^_*p*_ = 0.94, whereas young children showed the opposite (from 8.2 to 7.5%), *p* < 0.001, η^2^_*p*_ = 0.76. The effect of Preceding Choice-reaction/NoGo did not reach significance in older children, *p* > 0.9. RSI did not alter these trends (*p*s > 0.09).

#### Commission errors on NoGo trials

The transformed commission error rates were subjected to ANOVA with Age group (3), as a between-SS factor, and Preceding Choice-reaction/NoGo (2), and RSI (2), as within factors. The rate of commission errors decreased with advancing age (from 9.4, 5.6, and 3.9% for young children, older children, and adults, respectively, *F*_(2, 63)_ = 55.19, *p* < 0.001, η^2^_*p*_ = 0.64. The ANOVA yielded a significant effect of Preceding Choice-reaction/NoGo on the rate of commission errors, *F*_(1, 63)_ = 24.00, *p* < 0.001, η^2^_*p*_ = 0.28, but this effect was included in an interaction with Age group, *F*_(2, 63)_ = 6.19, *p* < 0.004, η^2^_*p*_ = 0.16. Moreover, the Age group × Preceding Choice-reaction/NoGo interaction was qualified by a significant higher-order interaction with RSI, *F*_(2, 63)_ = 42.76, *p* < 0.001, η^2^_*p*_ = 0.58. Separate ANOVAs were then performed on the data associated with each RSI. The Age group × Preceding Choice-reaction/NoGo (2) ANOVA done on the commission errors associated with RSI 50 ms yielded a significant interaction between the effects of Age group and Preceding Choice-reaction/NoGo, *F*_(2, 63)_ = 40, 0.36, *p* < 0.001, η^2^_*p*_ = 0.56. Further analyses indicated that the SCM effect reached significance in all three age groups, *p* < 0.001. Similar analyses done on the data associated with RSI 500 ms yielded a significant interaction between the effects of Age group and Preceding Choice-reaction/NoGo, *F*_(2, 63)_ = 9.67, *p* < 0.001, η^2^_*p*_ = 0.24, but the SCM effect was significant only for the child groups, *p*s < 0.001, not for the adults, *p* = 0.96.

In Table 3, it can be seen that for RSI 50 ms commission errors tended to decrease for NoGo-NoGo trial sequences relative to Choice-NoGo sequences in children, whereas this pattern was opposite for young adults. For RSI 500 ms, there was no sequential effect on the proportion of commission errors in adults while the pattern that can be observed for children seems opposite to the one associated with RSI 50 ms.

#### Response speed

Median RTs were subjected to ANOVA with Age group (3), as a between-SS factor, Preceding Choice-reaction/NoGo (2), and RSI (2), as within factors. The speed of responding increased with advancing age, *F*_(2, 63)_ = 448.33, *p* < 0.001, η^2^_*p*_ = 0.93. Adults (*M* = 433 ms) responded faster than older (*M* = 643 ms) and younger children (*M* = 812 ms). Responses were faster for RSI 500 (*M* = 602 ms) compared to RSI 50 ms (*M* = 656 ms); *F*_(1, 63)_ = 49.38, *p* < 0.001, η^2^_*p*_ = 0.44. This RSI effect did not interact with the effect of Age group, *p* > 0.25.

**Figure 4:**
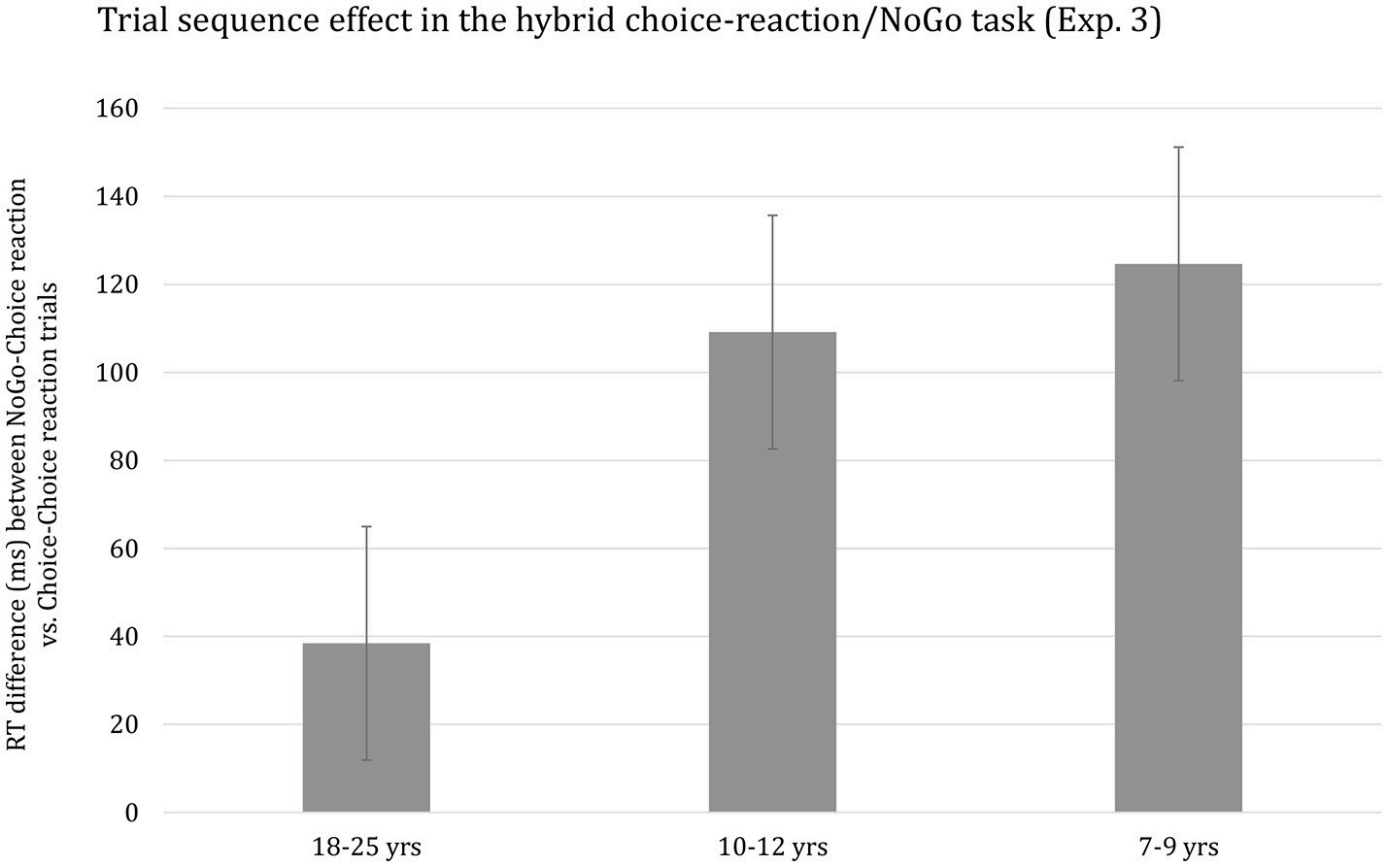
Reaction time difference (ms), including standard error bars, between NoGo-Choice Reaction vs. Choice-Reaction-Choice Reaction trials (i.e., Trial sequence effect) for each age group.

The effect of Preceding Choice-reaction/NoGo on median RT was highly significant, *F*_(1, 63)_ = 102.05, *p* < 0.001, η^2^_*p*_ = 0.62. Responses on Choice-reaction trials following a NoGo trial were considerably slower than when preceded by a Choice-reaction trial (*M* = 674 ms and *M* = 584 ms, respectively). The effect of Preceding Choice-reaction/NoGo interacted with the effect of Age group, *F*_(2, 63)_ = 8.62, *p* < 0.001, η^2^_*p*_ = 0.22. This interaction is plotted in Figure 4. It can be seen that the size of the Preceding Choice-reaction/NoGo effect observed for children almost triples the effect for adults. However, the developmental trend did not survive when using overall mean RT per participant as covariate, *p* > 0.29. Finally, the Preceding Choice-reaction/NoGo effect was somewhat larger for short (*M* = 97 ms) compared to long (*M* = 85 ms) RSI blocks, but this effect was far from significant, *p* > 0.53. Finally, the higher-order interaction including Age group, Preceding Go-NoGo, and RSI did not reach significance, *p* > 0.61.

As in the two previous experiments, we averaged data across the two RSIs and categorized trial sequences in terms of repetitions vs. alternations of arrow direction. The data were then submitted to ANOVA with Age group (3), as between SS factor and, Preceding Choice-reaction/NoGo (2), and Priming (2), as within-SS factors. The analysis yielded a highly significant interaction between the effects of Preceding Choice-reaction/NoGo and Priming, *F*_(1, 63)_ = 28.23, *p* < 0.001, η^2^_*p*_ = 0.31. For stimulus repetitions, the size of the SCM effect was 45 ms, whereas it was 136 ms for stimulus alternations. In Table 4, it can be seen that the difference between repetitions vs. alternations is due primarily to the fast responses on Go trials preceded by another Go trial when stimuli alternate. Follow-up analyses indicated that the SCM effect was significant for both stimulus repetition and alternation sequences (respectively, *p* < 0.003 and *p* < 0.001). Finally, the analysis revealed that the higher-order interaction including Age group was not significant, *p* = 0.16.

**Table 4:** Mean RT (ms) (incl. SD) for each trial sequence, age group (and overall), separated for repetition/alternation of arrow direction (Experiment 3).

**Trials sequences**				
	**Repetitions**		**Alternations**	
**Age group**	**Choice Reaction–Choice Reaction**	**NoGo–Choice Reaction**	**Choice Reaction–Choice Reaction**	**NoGo–Choice Reaction**
**RT/SD (ms)**				
7–9 years	799.08/159.93	858.16/118.16	700.31/100.08	890.53/58.98
10–12 years	628.95/32.66	691.40/83.30	547.01/68.42	702.83/74.47
18–25 years	424.07/41.13	438.47/28.75	402.52/39.79	464.96/32.13
Overall	617.37/93.36	662.68/84.01	549.95/72.71	686.11/58.63

*Choice Reaction-Choice Reaction, current Choice Reaction trial preceded by a Choice Reaction trial; NoGo-Choice Reaction, current Choice Reaction trial preceded by a NoGo trial; Choice Reaction-NoGo, current NoGo trial preceded by a Choice Reaction trial; NoGo-NoGo: current NoGo trial preceded by a NoGo trial*.

### Discussion

The current hybrid Choice-reaction/NoGo task required participants to perform a conjunction analysis involving the color and direction of the arrow stimulus. Thus, they were required to respond in the direction of the arrow, but only when the arrow was of a certain color, while they had to inhibit their response when the arrow was of a different color. The current findings indicated that, in spite of the requirement to perform a conjunction analysis, all age groups performed the task well, even the youngest children. Overall, both choice and commission error rates remained below 10%. The proportion of commission errors decreased with advancing age, consistent with notions suggesting that the ability to inhibit speeded responses increases when children are getting older (e.g., Casey et al., 1997; Jonkman et al., 2003; Cragg and Nation, 2008).

The speed of responding was delayed when choice-reaction trials were preceded by a NoGo trial relative to another choice-reaction trial. This finding is consistent with the Go/NoGo literature showing that responses on a Go trial are slowed when the Go trial follows response inhibition on a NoGo trial (e.g., Rieger and Gauggel, 1999; Hoffmann et al., 2003; Rieger et al., 2003; Schuch and Koch, 2003; Kleinsorge and Gajewski, 2004; Gade and Koch, 2005; Verbruggen and Logan, 2008; Jamadar et al., 2010). A cautionary note is in order here with regard to the current data. A straightforward comparison between the speed of responding on a choice-reaction trial preceded by another choice-reaction trial vs. a NoGo trial is hindered by a design issue that is difficult to avoid. We used an RSI of 50 or 500 ms but for a successful inhibit on NoGo trials there is no response. In that case, the interval from the NoGo stimulus to the stimulus on the subsequent trial is 3 s. Thus, the response delay on a Choice-reaction trial preceded by a NoGo trial relative to the response on a Choice-reaction trial preceded by another Choice-reaction trial could be due to the response vs. inhibit conflict on the preceding trial and/or the lengthening of the time interval between stimuli on successive trials. It should be noted, however, that a lengthening of the time-interval between trials is likely to result in faster responding due to enhanced preparation enabled by the longer interval (e.g., Näätänen et al., 1974; Adam et al., 2011). Accordingly, it seems fair to conclude that the current delay in the responding on a Choice-reaction trial preceded by a NoGo trial relative to another Choice-reaction trial is associated with SCM rather than preparation loss due to the lengthening of the time interval between successive stimuli.

SCM was present in all three age groups. There are few studies examining developmental change in the speed of responding following response inhibition on the immediately preceding trial. Consistent with the present findings, these studies revealed a developmental decrease in the delay of responding on Go trials preceded by a NoGo trial relative to the speed of responding on Go trials preceded by another Go trial. Thus, Huizinga and Van der Molen (2011; see also Van de Laar et al., 2011) examined the transition from a NoGo trial to a Choice-reaction trial and observed a pronounced delay on Choice-reaction trials following a NoGo trial for adults (about 60 ms) and this delay almost doubled for 11-year olds and increased close to 160 ms for 7-year olds. Huizinga and Van der Molen (2011) interpreted their data to suggest that the readiness to respond decreases following the encounter of a NoGo trial (see also Jamadar et al., 2010) resulting in an increase in response thresholds. The more pronounced delay in the speed of responding observed in children is then explained by assuming that adults are better able to fine-tune their response thresholds (cf. Huizinga and Van der Molen, 2011; p. 499).

In the current study, the developmental trend in SCM was even more sizeable than in Huizinga and Van der Molen (2011) study. The current findings showed that the size of the SCM effect was close to 40 ms adults and this effect basically tripled for the youngest children. However, this sizeable developmental trend did not survive when controlling for group differences in basic response speed suggesting that the mechanism implicated in SCM matures in concert with the other mechanisms involved in the translation of a stimulus into a response (e.g., Cerella and Hale, 1994; Kail and Salthouse, 1994). Finally, stimulus-repetition priming altered SCM but this change did not discriminate between age groups.

## General discussion

The current study set out to assess developmental change in SCM. In view of the literature indicating that SCM is domain specific (Braem et al., 2014), we employed three different tasks, as the developmental trend in SCM may depend on the type of conflict elicited by a task. In view of competing interpretations of SCM—top-down cognitive control vs. bottom-up repetition priming (e.g., Botvinick et al., 2001; Mayr et al., 2003)—we considered the potential influences of stimulus-repetition priming when analyzing the performance adjustments associated with SCM. We manipulated RSI, as it has been shown previously that SCM requires some time to manifest itself (Notebaert et al., 2006) and may die off when intervals are lengthened (Egner et al., 2010). Finally, we took care to assess the proportionality of the developmental trends in SCM, as the proportionality of developmental trends has important implications for interpretation (e.g., Cerella and Hale, 1994).

The current results showed a substantial congruency effect on the Simon task, the absence of a compatibility effect on the mixed SRC task, and commission errors on the hybrid Choice-reaction/NoGo task. These findings indicate that conflict is elicited on the Simon and hybrid Choice-reaction/NoGo task. On the SRC task, conflict is concealed because of the mixing of compatible and incompatible trials (e.g., Van Duren and Sanders, 1988; Stoffels, 1996). On the Simon task, participants responded to the color of the arrow while the direction of the arrow elicited a stereotypic tendency to respond into the direction of the arrow. Thus, on incongruent trials there is a conflict between responding in accordance with the color of the arrow vs. responding to the direction of the arrow (Lu and Proctor, 1995). On the SRC task, the color of the arrow informed participant which rule to apply when responding to the arrow direction. On half of the trials, the color of the arrow asked for compatible reactions (i.e., responding into the direction of the arrow). On the other half of the trials, the color of the arrow asked for incompatible reactions (i.e., responding opposite to the direction of the arrow). On those trials there is a conflict between the tendency to respond into the direction of the arrow and the task rule indicating that the opposite response is required (Proctor and Reeve, 1990). On the hybrid Choice-reaction/NoGo task, the color of the arrow told the participants which task rule should be applied. One color required participants to execute a binary choice reaction indicated by the direction of the arrow while the other color required participants to refrain from responding to the arrow. On those trials, there is a conflict between the tendency to respond elicited by trials requiring a choice reaction and the requirement to inhibit (e.g., Van Boxtel et al., 2001).

Children suffered more from conflict elicited by the tasks than adults. The size of the Simon effect decreased with advancing age, consistent with previous reports (e.g., Jerger et al., 1999; Davidson et al., 2006). Children committed more errors on NoGo trials of the hybrid Choice-reaction/NoGo task than adults. This observation is in accord with the extant literature on Go/NoGo tasks (Casey et al., 1997; Durston et al., 2002). Both patterns (i.e., greater Simon effect and more commission errors) have been interpreted to suggest that children experience greater difficulty in inhibiting pre-potent responses (Bjorklund and Harnishfeger, 1990; Dempster, 1992; Van der Molen, 2000), presumably because of the immaturity of the brain mechanisms involved in top-down inhibitory control (e.g., Rubia et al., 2006; Hwang et al., 2010; Crone and Ridderinkhof, 2011; Fjell et al., 2012). On the mixed SRC task, as anticipated, errors and RTs only slightly differed between compatible and incompatible reactions consistent with previous reports (e.g., De Jong, 1995; Jennings et al., 2002; Mansfield et al., 2012) but, surprisingly, this pattern did not discriminate between age groups. On the hypothesis that the absence of a compatibility effect on the speed and accuracy in the speed of responding when compatible and incompatible trials are mixed results from a preparatory strategy (i.e., on each trial, participants prepare for incompatible reactions), the current findings would suggest that children are equally able and inclined to adopt a preparatory strategy that, in addition, is as efficient as in adult participants. This interpretation is difficult to reconcile with notions suggesting a protracted developmental course of top-down cognitive control (e.g., Durston et al., 2006; Casey et al., 2008; Hwang et al., 2010; Munakata et al., 2012; Supekar and Menon, 2012; Astle et al., 2014).

All three tasks yielded SCM patterns but these patterns differed between tasks and were altered by the RSI manipulation. The results associated with the Simon task showed that the Simon effect was greatly reduced on trials preceded by incongruent trials relative to congruent trials. This finding is consistent with previous reports of SCM of the Simon effect (e.g., Soetens et al., 2010; Duthoo et al., 2014). Importantly, this pattern was observed only for trial sequences with a repetition of arrow direction, whereas the effect was basically annihilated for sequences with alternating arrow directions. This pattern suggests that SCM on the Simon task results primarily from bottom-up stimulus repetition priming. Thus, when specific stimulus (e.g., a RED arrow pointing to the LEFT) and response (e.g., execute a RIGHT response) features co-occur on a given trial they become bound together in episodic memory as an event-file (e.g., Hommel, 2004). When one of the stimulus features occurs on a subsequent trial (e.g., BLUE arrow pointing to the LEFT), the even-file is retrieved from episodic memory biasing the response system toward the response (execute a RIGHT response) that occurred on the previous trial (e.g., Logan, 1988).

The results associated with the SRC task showed that the SRC effect is greatly reduced or even reversed on trials preceded by an incompatible relative to a compatible trial, consistent with previous research (e.g., Jennings et al., 2002; Mansfield et al., 2012). This SCM effect was altered by RSI—the effect was more pronounced for the short relative to the long RSI. Stimulus repetition priming failed to influence the SCM effect. Previous reports attributed the reduction or reversal of the SRC compatibility effect on trials preceded by an incompatible trial to a preparatory strategy favoring incompatible responses (e.g., De Jong, 1995; Jennings et al., 2002). This interpretation would be compatible with the current lack of a bottom-up stimulus repetition influence but, at the same time, it is difficult to reconcile with the observation that the SCM effect is larger for the short compared to the long RSI. A unified account of the current pattern of results might be provided by resorting to the task-switching literature (Vandierendonck et al., 2010). This literature shows that switching between task sets involves a cost and costs are higher when switching from a difficult to an easy task compared to switching from an easy to difficult task. Switching costs have been attributed to the persisting activation of the previous task set, which interferes with the activation of the appropriate task set on the current trial (e.g., Allport et al., 1994). Thus, the incompatible mapping rule used on the previous trial (i.e., the difficult task set) interferes with the activation of the compatible mapping rule (i.e., the easy task set) that is required on the current trial, which results in a considerable delay in the speed of responding. In contrast, the persisting incompatible mapping rule facilitates responding on the current trial when an incompatible response is called for. The current observation that the SCM effect was more pronounced when RSI was short compared to long can then be explained by assuming that the activation of task sets dissipates over time.

The findings that emerged from the hybrid Choice-reaction/NoGo task showed a pronounced delay on choice-reaction trials that followed a NoGo trial relative to another choice-reaction trial. In contrast to the data associated with the mixed SRC task, the effect was not altered significantly by RSI and, in contrast to the data associated with the Simon task, it was relatively more pronounced for alternations of arrow direction relative to repetitions. This effect was primarily due to faster responding on alternating arrow direction trials relative to trials associated with arrow direction repeats. Previous research indicated that the typical repetition benefit might change into an alternation benefit when stimulus features relevant to the task change across trials. That might have happened here. Trials were sorted for arrow direction (indicating which response should be selected) but the color of the arrow (indicating whether a response should be executed) varied across trials (Kleinsorge, 1999). Importantly, however, the SCM effect reached significance for both arrow-direction alternation and repetition trials indicating that the effect cannot be fully attributed to stimulus repetition influences. The current findings could be interpreted along similar lines as the data from the SRC task. That is, the task set of the previous trial (e.g., INHIBIT) persists and interferes with the task set required on the current trial (e.g., EXECUTE CHOICE REACTION). It should be noted, however, that the Choice-reaction/NoGo task did not require the selection of one out of two task rules depending on the color of the arrow as in the SRC task but rather a conjunction analysis of arrow direction, needed for the selection of a response, and arrow color, needed for determining whether the selected response should be executed or not. One of the task-relevant features of the stimulus (either direction or color) might be associated with the outcome of the conjunction analysis on the previous trial (e.g., INHIBIT) and when this feature re-occurs on the current trial the outcome (INHIBIT) is retrieved from memory delaying a response when response execution would be the appropriate outcome of the current conjunction analysis (e.g., Verbruggen and Logan, 2008).

The primary goal of the current study was to assess age-related changes in the SCM effect. One important finding is that SCM was seen in the youngest children on all three tasks. This finding is consistent with previous research showing that young children delay responding on trials after encountering conflict on the preceding trial (Stins et al., 2007; Huizinga and Van der Molen, 2011; Van de Laar et al., 2011; Larson et al., 2012; Iani et al., 2014; Ambrosi et al., 2016; Cragg, 2016). This observation has been interpreted to suggest that even young children adapt after conflict (e.g., Larson et al., 2012) and, more specifically, that the immature brain of children does not prevent them to balance pro- and reactive control in the face of conflict (e.g., Ambrosi et al., 2016). Such an interpretation, however, begs the question of how young children manage this intricate balancing of pro- and reactive control given the protracted developmental course of the neural mechanisms implicated in conflict adaptation (for a review Crone and Steinbeis, 2017).

The present results yielded a developmental decrease in the size of the SCM effect on all three tasks, although the exact pattern differed between tasks. A developmental decrease is consistent with previous reports of age-related changes in the speed of responding following conflict (Huizinga and Van der Molen, 2011; Van de Laar et al., 2011; Larson et al., 2012; Araujo et al., 2015; Cragg, 2016; Smulders et al., 2016; but see Waxer and Morton, 2011). The results obtained using a Simon task are consistent with the findings reported previously by Araujo et al. (2015). The current findings indicated that the developmental trend occurred only for the short, not the long RSI. In addition, the developmental trend occurred only for trial sequences associated with stimulus repetitions not for alternation sequences. This pattern of results suggests that the developmental trend in the SCM effect on the Simon task results from bottom-up associative priming rather than top-down cognitive control.

The developmental trend of the SCM effect was not altered by RSI or stimulus repetition. The SCM effect on the SRC task was interpreted in terms of the interference of the task set associated with the previous trial with the activation of the task set required on the current trial. A developmental decrease in the size of the SCM effect may then suggest that young children are more susceptible to the task set interference. Indeed, previous studies reported that young children experience greater interference from previous task rules (Cepeda et al., 2001; Crone et al., 2006; Gupta et al., 2009; Kray et al., 2012; Weeda et al., 2014).

The results obtained from the hybrid Choice-reaction/NoGo task yielded a pronounced developmental decrease in the size of the SCM effect consistent with the findings reported by Araujo et al. (2015). The developmental trend of the SCM effect obtained using the Choice-reaction/NoGo task was not affected by RSI and stimulus repetition. The SCM effect on this task was interpreted in terms of an automatic retrieval from memory of the outcome of the conjunction analysis triggered by stimulus feature overlap across successive trials. This notion has been adopted from Verbruggen and Logan (2008) who suggested that, once a stimulus (feature) has been consistently linked to the need to inhibit a response, this stimulus (feature) will automatically activate the inhibition goal from memory and thus promote the required response inhibition without intervening top-down processes (see also Verbruggen et al., 2014). This interpretation is consistent with the idea that proactive top-down control is costly and, thus, most individuals opt for avoiding it (e.g., Dunn et al., 2016) whenever possible while it is beyond the power of most young children (e.g., Munakata et al., 2012).

A final feature of the current data must be discussed. Although the data showed a developmental decrease in the SCM effect for all three tasks, the subsequent analysis considering age group differences in the global speed of responding revealed that the developmental trends did not survive. This result suggests that the mechanisms involved in the SCM effect mature at a similar rate as the other mechanisms comprised in the translation of a stimulus into a speeded response (e.g., Cerella and Hale, 1994). It is then important to ask which mechanisms are subject to bottom-up control, conflict-triggered control. Bisset and Logan (2011) considered this question within the context of stochastic accumulator models (e.g., Ratcliff and Rouder, 1998; Usher and McClelland, 2001; Brody and Hanks, 2016). These models assume that stimulus processing consists of a noisy accumulation of evidence over time. When the evidence is hitting a predefined boundary, a response is emitted. Within this context, the choice reaction process is described in terms of a set of four parameters; the onset of accumulation, the rate of accumulation, the threshold of accumulation and a parameter, non-decision time, referring to stimulus encoding and response execution. Previously, we applied diffusion modeling to the data derived from a developmental task-switching study (Weeda et al., 2014). The results showed that non-decision time was considerable prolonged on task-switch relative to repeat trials and the size of this effect decreased with advancing age (see also Janczyk et al., 2017, who used a cross-talk paradigm). The lengthening of non-decision time on switch trials was interpreted to suggest in terms of a delay in the retrieval of the current task set from memory and this delay might be more pronounced for young children (e.g., Dionne and Cadoret, 2013). In addition, it was found that drift rate increased with task-set repetitions and this trend was more pronounced for young children relative to the older participants. The observation that drift-rate was lowest on the first task-set repetition was interpreted to suggest interference from previous task set, which might be stronger for young children (e.g., Crone et al., 2006; Gupta et al., 2009). Finally, Weeda et al. (2014) observed a substantial developmental decrease in threshold separation indicating that young children adopt a more conservative response criterion than adults (see also Ratcliff et al., 2012). But this developmental trend did not differentiate between task-switch and repeat trials (see also Schmitz and Voss, 2014). Collectively, the results obtained by Weeda et al. (2014) are compatible with the idea that the effect of task switching on choice reactions is related to bottom-up control mechanisms. Further investigations of the mechanisms that mediate control in post-conflict performance would be an important goal for future research.

This study has at least two limitations. First, an important goal of the current study was to examine developmental change in SCM across different conflict tasks. As previous research demonstrated the domain specificity of SCM (e.g., Braem et al., 2014), we wondered whether developmental change in SCM would be typified by the nature of the conflict that is encountered. A limitation of the current study is then the between-group design that was used. For each of the three conflict tasks examined in the current study, we recruited different groups of participants. This was done to obtain a sufficient number of observations for each trial × sequence combination per task and for each group, including the youngest children. Ideally, the comparison of developmental change in SCM across tasks should have been done using a within-group design but we did not want to bury the youngest children under a mountain of trials. A possible solution would be to run a series of simulations to assess the number of trials needed for each trial × sequence type analogous to the simulations performed by Band and co-workers when examining the amount of trials needed for obtaining robust and reliable stop-times using a stop-signal task (Band et al., 2003).

A second, but related, limitation refers to the current, only partial, analysis of repetition effects vis-à-vis SCM. We considered only arrow-direction repetitions and ignored response repetitions, as we did not have a sufficient number of trials for examining both. It should be noted, however, that response-related effects were included automatically when selecting trials for arrow-direction as can be seen in the Appendix in Supplementary Materials. Thus, for the Simon task Congruent-Congruent sequences, repetition of arrow direction was associated with response repetition and alternation of arrow direction with response alternation. The same applies to Incongruent-Incongruent sequences. In addition, for both Congruent-Incongruent and Incongruent-Congruent sequences, repetition of arrow direction is associated with response alternation whereas alternation of arrow direction is associated with response repetition. Accordingly, we do not expect to obtain different SCM patterns when selecting trials for bot arrow direction repetition *and* response repetition. For the SRC task, Compatible-Compatible and Incompatible-Incompatible sequences consist of either arrow-direction and response repetitions or arrow-direction and response alternations. For the two other type of sequences, arrow-direction repetitions are associated with response alternations or vice-versa. Consequently, selecting trials for both arrow-direction *and* response repetition or arrow-direction *and* response alternation may add somewhat more precision but is not likely to alter the current pattern of results. Finally, for the hybrid Choice/GoNoGo task, there are only two types of sequences to be considered—Choice-Choice and NoGo-Choice. Responses are not obtained for the two other types of sequences. For the Choice-Choice sequences, arrow-direction repetition is associated with response repetition and arrow-direction alternation with response alternation. For the NoGo-Choice sequences there is only repetition or alternation of response side in addition to the repetition or alternation of arrow direction (i.e., the side of response inhibition on NoGo trials and the side of responding on Choice trials). For these sequences, arrow-direction repetition is associated with response (side) repetition and arrow-direction alternation with response (side) alternation. Again, it is unlikely that selecting for both arrow-direction *and* response (side) repetitions or alternations would have resulted in a substantially different pattern of results. That being said, it would have been more elegant to select for both stimulus and response repetitions, given sufficient trial numbers, to assess the specific contribution of each repetition sequence to the observed patterns of SCM effects (e.g., Nieuwenhuis et al., 2006). Even better, experimental designs could have been used that allow for a priori control of repetition priming effects (e.g., Duthoo et al., 2014). Interestingly, the use of such designs seem to demonstrate that SCM may occur in the absence repetition priming effects (e.g., Kim and Cho, 2014; Schmidt and Weissman, 2014). An interesting challenge for future research is to assess how developmental change in conflict modulation is altered when removing the potential contribution of repetition priming.

## Conclusion

The current study yielded three major findings. First, SCM was present already in young children. We argued that this finding should not be interpreted to suggest that young children are able to adjust their performance following conflict by exercising some sort of top-down cognitive control. In contrast, collectively, our findings suggested that the current SCM effects should be interpreted in terms of bottom-up control mechanisms. Secondly, the size of the SCM effects decreased with advancing age but the downward trends did not survive when considering age-group differences in global response speed. This finding indicates that the mechanisms implicated in SCM and the other mechanisms included in the choice reaction process are not differentially sensitive to the effects of advancing age. Thirdly, the results showed that post-conflict performance varied across tasks. The Simon task yielded a substantial congruency conflict and post-conflict effects were observed only for stimulus repetition sequences. The mixed SRC task yielded a reversal of the typical SRC effect and post-conflict effects were not altered by RSI or stimulus repetitions. The hybrid Choice reaction/NoGo task showed a considerable delay in the speed of responding on trials following a NoGo trial and this effect was altered by stimulus repetitions but not RSI. Overall, the current pattern of results suggests that the type of performance conflict is dissimilar across tasks and that post-performance conflict is, most likely, mediated by multiple mechanisms that are differentially sensitive to advancing age. The current study was only scratching the surface. Much remains to be learned in the domain of developmental change in SCM.

## Ethics statement

This study was carried out in accordance with the recommendations of Faculty Ethics Review Board (FMG-UvA) and with written informed consent from all subjects. All subjects gave written informed consent in accordance with the Declaration of Helsinki. The protocol was approved by the Faculty Ethics Review Board of the Faculty of Social and Behavioral Sciences (ERB).

## Author contributions

All authors listed have made a substantial, direct and intellectual contribution to the work, and approved it for publication.

### Conflict of interest statement

The authors declare that the research was conducted in the absence of any commercial or financial relationships that could be construed as a potential conflict of interest.
