# Supplementary material for: How Do Children Deal With Conflict? A Developmental Study of Sequential Conflict Modulation
Source: Front Psychol. 2018 May 23;9:766. doi: 10.3389/fpsyg.2018.00766 (PMC5974159; doi:10.3389/fpsyg.2018.00766)
Supplement: Supplementary file 1 [file Data_Sheet_1.pdf]

<u>Appendix</u>: All possible sequences for each conflict task.

Simon task: Respond to color of left vs. right pointing arrows (e.g., red is left and blue is right).

Sequences:			
**C-C**	**I-I**	**C-I**	**I-C**
red arrow left - response left<br>red arrow left - response left (CR)	red arrow right - response left<br>red arrow right - response left (CR)	red arrow left - response left<br>red arrow right - response left (CR-DA-RR)	red arrow right - response left<br>red arrow left - response left (CR-DA-RR)
blue arrow right - response right<br>blue arrow right - response right (CR)	blue arrow left - response right<br>blue arrow left - response right (CR)	blue arrow right - response right<br>blue arrow left - response right (CR-DA-RR)	blue arrow left - response right<br>blue arrow right - response right (CR-DA-RR)
red arrow left - response left<br>blue arrow right - response right (CA)	red arrow right - response left<br>blue arrow left - response right (CA)	red arrow left - response left<br>blue arrow left - response right (CA-DR-RA)	red arrow right - response left<br>blue arrow right- response right (CA-DR-RA)
blue arrow right - response right<br>red arrow left - response left (CA)	blue arrow left - response right<br>red arrow right - response left (CA)	blue arrow right - response right<br>red arrow right - response left (CA-DR-RA)	blue arrow left - response right<br>red arrow left - response left (CA-DR-RA)

C-C and I-I sequences are either complete repetitions or sequences alternating both arrow direction AND responses. C-I and I-C are sequences where arrow direction and response side correlate 0%.

Appendix (continued)

S-R Compatibility task: Respond to arrow direction when blue and opposite when red.

Sequences:			
**C-C**	**I-I**	**C-I**	**I-C**
blue arrow left - response left	red arrow left - response right	blue arrow left - response left	red arrow left - response right
blue arrow left - response left (CR)	red arrow left - response right (CR)	red arrow left - response right (CA-DR-RA)	blue arrow left- respons left (CA-DR-RA)
blue arrow right - response right	red arrow right - response left	blue arrow right - response right	red arrow left -response right
blue arrow right - response right (CR)	red arrow right - response left (CR)	red arrow left - response right (CA-DA-RR)	blue arrow right - response right (CA-DA-RR)
blue arrow left - response left	red arrow left - response right	blue arrow left - response left	red arrow right - response left
blue arrow right - response right (CR-DA-RA)	red arrow right - response left (CR-DA-RA)	red arrow right - response left (CA-DA-RR)	blue arrow left - response left (CA-DA-RR)
blue arrow right - response right	red arrow right - response left	blue arrow right - response right	red arrow right - response left
blue arrow left - response left (CR-DA-RA)	red arrow left - response right (CR-DA-RA)	red arrow right - response left (CA-DR-RA)	blue arrow right- response right (CA-DR-RA)

C-C and I-I sequences are either complete repetitions or sequences alternating both arrow direction AND responses. C-I and I-C are sequences where arrow direction AND responses alternate only 50% of the time.

Appendix (continued)

Hybrid Choice/NoGo task: Respond right to red arrows pointing to the right and left to blue arrows pointing to the left. Refrain from responding to red arrows pointing to the left or blue arrows to the right.

Sequences:			
**Choice-Choice**	**NoGo-NoGo**	**Choice-NoGo**	**NoGo-Choice**
red arrow right - response right<br>red arrow right - response right (CR)	No reaction times	No reaction times	red arrow left - no left response<br>red arrow right - response right (CR-DA-RA)
blue arrows left - response left<br>blue arrows left - response lef (CR)			blue arrow right - no right response<br>blue arrow left - response left (CR-DA-RA)
red arrow right - response right<br>blue arrows left - response left (CA)			red arrow left - no left response<br>blue arrow left - left response (CA-DR-RR)
blue arrows left - response left<br>red arrow right - response right (CA)			blue arrow right - no right response<br>left arrow right - right response (CA-DR-RR)

C-C sequences are either complete repetitions or alternations. I-C sequences are only partial repeats (color but not direction of the arrow or arrow direction but not color) and arrow direction and response side correlate 100%.

Note: CR; color repetition. CA; color alternation. DR; direction repetition. DA; direction alternation. RR; response repetition. RA; response alternation
